# Primary stroke prevention in China – a new approach

**DOI:** 10.1179/1743132815Y.0000000025

**Published:** 2015-05

**Authors:** Valery L. Feigin, Wenzhi Wang, Hua Fu, Liping Liu, Rita Krishnamurthi, Rohit Bhattacharjee, Priya Parmar, Tasleem Hussein, Suzanne Barker-Collo

**Affiliations:** 1 National Institute for Stroke and Applied Neurosciences at AUT University, Auckland, New Zealand; 2 Department of Neuroepidemiology, Beijing Neurosurgical Institute, People's Republic of China; 3 Health Communication Institute, Fudan University, Shanghai, People's Republic of China; 4 Beijing Tiantan Hospital, Capital Medical University, Beijing, People's Republic of China; 5 Information and Communication Technology Services at AUT University, Auckland, New Zealand; 6 School of Psychology, The University of Auckland, Auckland, New Zealand

**Keywords:** stroke, prevention, Stroke Riskometer App, China

## Abstract

The growing burden of stroke in China, along with the increasing cost of health care calls for new, more effective strategies for stroke prevention. These strategies should include increasing awareness of stroke symptoms, awareness of risk factors, and provision of easily available information on means of modifying risk factors. The Stroke Riskometer App is exactly such a tool, available in Mandarin, for adult individuals to calculate their risk of stroke over the next 5 and 10 years, and to identify their individual stroke risk factors and linking them to possible means of modifying these risk factors. The use of this App could reduce the risk of stroke for individuals in the Chinese population and contribute to significant reduction in stroke burden in China.

In 2010, stroke remained the first leading cause of death and became the second leading cause of life years lost in China.^1^ Although a combination of mass and high-risk approaches for stroke prevention showed encouraging effects among the Chinese population,^2^,^3^ the stroke burden in China continues to increase (Fig. 1) over the last two decades,^4^,^5^ suggesting that primary stroke prevention strategies are not well-translated into practice.^5^–^7^ To bridge the gap between guidelines and practice and increase medical resource investments in China, there is an urgent need to improve individual knowledge on primary stroke prevention^6^ and to have an effective screening tool for selecting Chinese people at high risk of stroke.^8^

**Figure 1:**
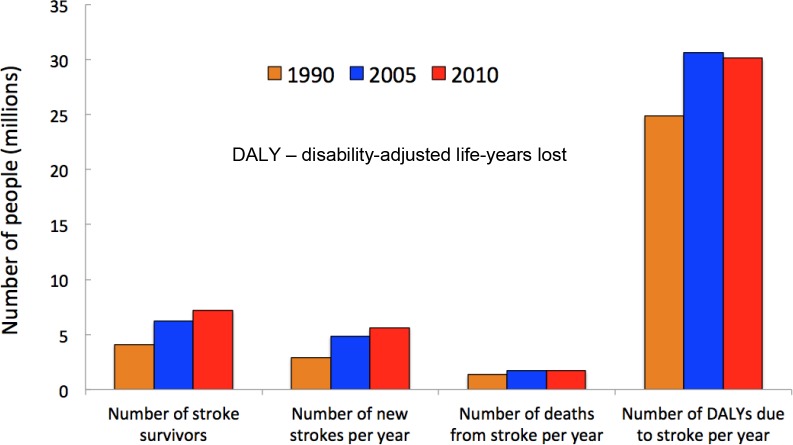
Trends in stroke burden in China in 1990–2010: GBD 2010 Study.

The National Institute for Stroke and Applied Neurosciences at AUT University in collaboration with AUT Enterprise Limited and New Zealand Stroke Education (charitable) Trust has recently developed the Stroke Riskometer^TM^ App (Fig. 2), translated into 12 of the world's most spoken languages. The translation of the App into Mandarin has been done in collaboration with and under direction of Prof. Wenzhi Wang (Beijing Institute of Neurosurgery) and Prof. Hua Fu (Health Communication Institute, Fudan University). The App calculates the 5-year and 10-year risk of stroke in individuals aged 20 years and over. This is a personalised assessment where individuals identify their own risk factors associated with stroke. There are 19 risk factors included in the App: age, sex, race/ethnicity, weight and height to calculate Body Mass Index, smoking, alcohol and fruit/vegetable consumption, physical activity, stress, family history of stroke or heart attack, systolic blood pressure, blood pressure lowering medication, as well as the presence of diabetes mellitus, heart or peripheral artery disease, history of left ventricular hypertrophy, atrial fibrillation, dementia or cognitive problems, traumatic brain injury, and previous stroke or transient ischaemic attack. Answering all the questions in the App takes only about 2 minutes and does not require any laboratory tests. Uniquely, the App provides estimates of not only absolute but also relative risk of stroke; therefore, allowing the users to compare their risk of having a stroke against someone of their age and sex without their individual additional risk factors, thus representing a new paradigm in primary stroke prevention.^9^ This is particularly important for Chinese people at low to moderate risk of having a stroke who are currently not covered by high-risk stroke prevention strategies and who constitute over 80% of people who develop a stroke later in the life. Using the App, these people can be motivated to control their risk factors and reduce their risk of having a stroke. As many of the risk factors for stroke also increase risk of other health issues, this has the added potential of reducing the risk of heart attack, dementia, and diabetes mellitus. The Stroke Riskometer App has been validated^10^ against two commonly used stroke prediction algorithms (Framingham and QStroke) and endorsed by the World Stroke Organisation, World Federation of Neurology, and International Association on Neurology and Epidemiology. In order to be accessible to as many people as possible, the Stroke Riskometer App has been translated into 11 most commonly spoken languages (Mandarin, Hindi, Spanish, Russian, Arabic, Bengali, Portuguese, Malay, French, German, Japanese) covering over 160 countries (5.6 billion people). The smartphone-based platform means that the potential reach of this app is enormous. With approximately 1.75 billion people in the world owning smartphones,^11^ people across the globe will have easy access to their stroke risk and risk factors in their own languages.

**Figure 2:**
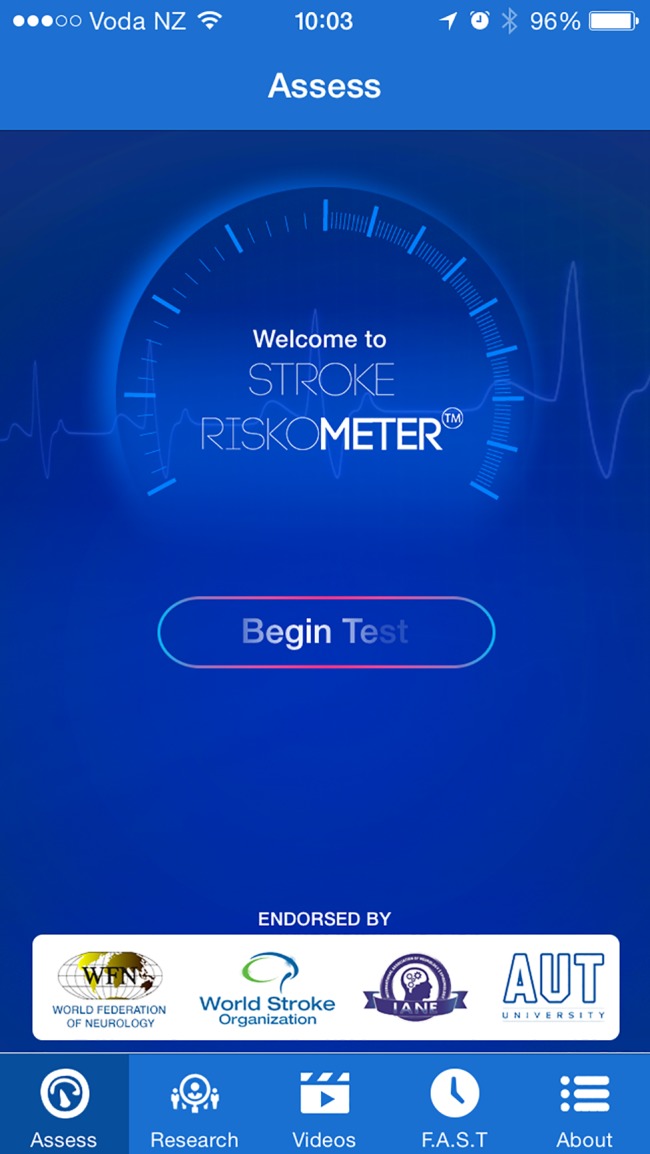
Stroke Riskometer App^TM^

Apart from empowering the user to take some control of their health, the App educates people about the warning signs of stroke and provides evidence-based recommendations for controlling their risk factors (including educational video clips). These educational materials and recommendations are based on internationally accepted guidelines for primary stroke and cardiovascular disease prevention. The App users have an option to save their results and monitor their progress in terms of stroke prevention. They can also email their results to a doctor or another person of their choice. However, the App should not be considered as a substitute for doctor's advice but rather as an adjunct, educational and motivational tool for individuals and health service providers. This will step towards bridging the gap between guidelines and practice and increasing medical resource investments in China. Furthermore, as the App is available on both iOS (iPhones, iPads) and Android platforms, it has the potential to be used by about 700 million Chinese smartphone users, including rural people who may have a limited access to health care facilities. Preliminary evidence suggests that the app appears to be appealing to the individuals concerned, as it empowers them to know and self-manage their risk and risk factors. Regular and wide use of this app could be as efficient as the conventional population-based approach, because it allows identification and engagement in prevention of all individuals who are at even slightly increased risk of stroke and cardiovascular disease. We also encourage health professionals to use the app in their everyday practice.

Another important application of the Stroke Riskometer App in China (and internationally, potentially covering over 160 countries) is its use as a research tool for epidemiological and interventional studies (cross-sectional, prospective cohort studies, and randomised controlled trials) on primary and secondary prevention of major non-communicable diseases (NCDs), such as stroke, ischaemic heart disease, cognitive impairment, dementia, and diabetes mellitus. The Ethics Committee of the AUT University has approved this research component of the App. All users of the App will be offered an opportunity to participate in the research and only data from those who consent to participate in the study will have their data transferred to the central database of the Coordinating Centre (AUT University, Auckland, New Zealand). All these data will be anonymised and encrypted to ensure confidentiality of the data, and all study participants will have an option to withdraw from the study at any time. The Information Technology Systems for research data collection, storage and validation are currently being developed at AUT University. Research data collected during the cross-sectional (prevalence) and cohort studies will allow the refinement of the stroke prediction algorithm and development of algorithms for prediction of stroke, ischaemic heart disease, dementia, and diabetes mellitus specific to the Chinese population. Further validation of the algorithms for the Chinese population is also planned. It is expected that the implementation of the Stroke Riskometer App will contribute to the reduction of the burden of NCD in China.

## Conclusion

Effective primary stroke prevention is the only solution to reduce stroke burden; but currently used primary stroke prevention strategies are not effective enough in China, because stroke burden there continues to increase. With recent advances in telecommunications and mobile technologies (smartphones), the Stroke Riskometer app will allow for individualised primary prevention of stroke and associated health conditions, which has the potential to reach a large proportion of the Chinese population and reduce the burden of NCD in China.

## Disclaimer Statements

**Contributors** Valery L. Feigin: designed the study and wrote the first draft. Wenzhi Wang: contributed to the critical revision of the manuscript for important intellectual content and contributed to the development of the Chinese version of the App. Hua Fu, Liping Liu, Rita Krishnamurthi, Rohit Bhattacharjee, Priya Parmar, Tasleem Hussein, Suzanne Barker-Collo: contributed to the critical revision of the manuscript for important intellectual content.

**Funding** The Faculty of Health and Environmental Studies, Auckland University of Technology (AUT), AUTEL, and AUT University Research Office, Auckland, New Zealand. Co-authors from the AUT University (Valery L. Feigin, Rita Krishnamurthi, Rohit Bhattacharjee, Priya Parmar, Tasleem Hussein) declare that funds resulting from the sale of the professional version of the Stroke Riskometer app go into further research and education for stroke prevention. None of the other authors has competing financial interests.

**Conflicts of interest** Co-authors from the AUT University (VLF, RK, RB, PP, TH) declare that funds resulting from the sale of the professional version of the Stroke RiskometerTM app go into further research and education for stroke prevention. None of the other authors has competing financial interests.

**Ethics approval** The Ethics Committee of the AUT University has approved this research component of the Stroke Riskometer App.
